# Gender Differences in the Level of Achievement of Gymnastic and Acrobatic Skills

**DOI:** 10.3390/ijerph17197216

**Published:** 2020-10-02

**Authors:** M.ª Alejandra Ávalos-Ramos, Lilyan Vega-Ramírez

**Affiliations:** Department of General and Specific Didactics, Faculty of Education, University of Alicante, 03690 Alicante, Spain; sandra.avalos@ua.es

**Keywords:** university students, gymnastics skills, motor learning, physical education

## Abstract

Physical education should provide the opportunities to progressively integrate motor tasks with different levels of complexity from early ages to adolescence. The objective of this research was to analyze gender differences in the level of achievement of basic acrobatic skills of students of physical activity sciences in their non-university stages. This cross-sectional study with descriptive design was carried out with a total of 675 first-year Spanish university students distributed over eight academic years. An initial test of two acrobatic skills was performed. The instruments used for data collection were a video camera and observation templates. The SPSS 26.0 computer program was used for data analysis. The main results show a poor and insufficient level of technical achievement by both genders, but more pronounced for men. The most deficient phases were those related to extension movements of different segments of the body and movements that require coordination and stability in the descent phases of acrobatics, and men were less flexible in both phases. Non-university training of the content associated with basic gymnastic and acrobatic skills is being deficient within the subject of physical education with a downward trend of not only the development of sports skills but also of physical abilities such as flexibility and strength.

## 1. Introduction

The practice of different sports disciplines associated with gymnastics and acrobatic skills such as artistic, rhythmic and acrobatic gymnastics, among others, involves developing a series of exercises that require components of strength, flexibility, agility and coordination, thus providing countless benefits. In this vein, the usual practice of gymnastics by boys and girls contributes to improving postural control in bipedal positions, where stability and orientation skills become very important in the development of the individual, along with locomotive and object control skills [1]. Practicing gymnastic and acrobatic skills at an early age will create a background of motor exercises in situations of rotation, body investment and body sustainability, using all body segments. Rudd et al. [2] showed that the implementation of an eight-week gymnastics program for children significantly improved overall coordination, fundamental movement skills and physical self-concept compared to students who continued with the standard physical education program in which gymnastic activity was not present. In addition, significant improvements in abdominal strength, flexibility, aerobic fitness and strength have been demonstrated after gymnastics programs were implemented for children and adolescents during 12 weeks of intervention [3].

Motor learning is considered to be the set of internal processes related to practice, and motor experience should lead to relatively lasting changes in the ability to produce motor activities, not being considered an aide to short-term changes [4,5,6]. A well-learned skill is maintained over time after a period without practice [5,7], and in gymnastics practice is imperative [8]. There are many factors that can influence motor learning and the learning of gymnastic skills. In this sense, it is necessary to develop basic skills in order to later introduce more complex elements, and it is essential to control the amount and variety of gymnastic experiences in physical education classes in order to adequately learn these skills. In addition, aspects associated with students such as motivation, a predisposition towards the practice the initial level of learning skill and/or the feedback received can be decisive in the learning process [9]. In the case of interests in the practice of physical and sports activity, this is different depending on gender. In relation to participation in after-school sports, for example, girls are less involved than boys, with the curve declining mainly from adolescence [10,11]. Boys practice more, for a longer time and do so in the company of others, opting for cooperative or competitive activities in the framework of competitions and tournaments [12]. Instead, girls are more drawn to individual sports associated with disciplines such as dance, gymnastics or skating and set long-term goals [13].

Another determining factor in learning gymnastic skills is the influence it has on the selection of content to be developed in teaching programs within the subject of physical education, as well as strategies used for learning and the form of communication [14,15]. In countries such as the United States and the United Kingdom, content developed in physical education classes often favors the interests and preferences of boys rather than girls, which is harmful in terms of participation in and motivation for activities proposed by teachers [16]. It is important to note that teaching programs in physical education curriculum in the secondary education stage focus on developing and acquiring sports skills, among others [17]. In this line, gymnastic and acrobatic skills are directly and indirectly integrated into the curriculums of different educational stages, such as in Spain in primary education, compulsory secondary education and baccalaureate and in the educational systems of countries such as England, Germany and Canada [18,19].

Considering all the above, the aim of this study was to identify and analyze gender differences from eight academic years different in the level achieved in two basic acrobatic skills after previous stages of training by first-year students in the Sciences of Physical Activity and Sport (SPAS).

## 2. Materials and Methods

This cross-sectional, quantitative and descriptive study included 675 volunteer participants (501 men and 174 women) selected for convenience and availability, aged 18–34, with a mean age of 20.2 years (SD = 3.638; women aged 18–28 with a mean age of 19.52, SD = 2.400; men aged 18–34 with a mean age of 20.48, SD = 3.948). The students were informed that the data from the initial evaluation could be used for research purposes, obtaining informed consent, following the guidelines of the data projection law and the approval of the Ethics Committee of the University of Alicante (UA-2020-08-29).

Students were enrolled from academic year 2010–2011 to 2017–2018 in the gymnastic and artistic skills class, part of the first course of the SPAS degree at a Spanish university (Table 1).

### 2.1. Instrument

The instruments used for diagnostic evaluation of skill were a video camera and two observation templates [20,21], which were designed to evaluate two basic stunts: cartwheel and handstand. The templates described the technical phases and specified the assignment of a numeric value according to the importance of the phase of movement, with a total value of 10 points for the acrobatic element. A pilot test was conducted with 50 students, where two expert gymnastics evaluators contrasted the results recorded on the students’ observation templates. To check the internal reliability of the proposed instruments, Cronbach’s alpha coefficient was calculated for each skill, with the following results: cartwheel alpha = 0.71 and handstand alpha = 0.85.

### 2.2. Cartwheel Phases

1. Wide step forward: up to 1 point
2. Alternate hand support: up to 2 points
3. Legs open and straight down the vertical: up to 4 points
4. Alternate foot support: up to 2 points
5. Finish standing and extend arms: up to 1 point
6. Does not perform the skill: 0 points

### 2.3. Handstand Phases

1. Hands support away from the forward foot: up to 1 point
2. Simultaneous support of hands on the ground and separated at shoulder height: up to 1 point
3. Extended body alignment of the trunk–arm segments: up to 2 points
4. Extended body alignment of the arm–trunk–leg segments: up to 2 points
5. Keeps the inverted position for 2 s without help: up to 3 points
6. Reception of alternate legs: up to 1 point
7. Does not perform the skill: 0 Points

In addition, the overall achievement level was established as follow: outstanding (OS; 9–10 points), remarkable (RM; 7–8 points), not bad (NB; 6 points), enough (EN; 5 points), insufficient (INS; 3–4 points), poor (P; 1–2 points), very poor (VP; 0 points) and does not perform the acrobatic movement (NE).

### 2.4. Procedure

After the observation templates for the diagnostic evaluation were designed, all students in the initial session took a technical execution test where they had two attempts to perform the corresponding stunts while being assessed for technical execution. The protocol carried out during the eight academic years (from 2010–2011 to 2017–2018) was analyzed as follows:

- Students were informed about the need to take a diagnostic test, the content of the test and its recording procedure.
- There was a 10-min specific warm-up period prior to the test.
- Students were individually evaluated in the technical test.
- The professors in situ carried out the evaluation, with support in the observational templates.
- The professors and two professors who specialized in gymnastics reviewed and subsequently evaluated the recordings of the technical test.

### 2.5. Data Analysis

IBM SPSS 26.0 for Windows software was used for data analysis, applying descriptive statistics across cross-tables and percentages. For verification of the normality of the sample, the Kolmogorov–Smirnov test was used, and, for comparison of nonparametric variables of ordinal data, the Mann–Whitney U-test and chi^2^ (*X*^2^) were used; a significance level of *p* ≤ 0.05 was established.

## 3. Results

Hereafter, we explain the results obtained after analyzing the tests, which are expressed in percentages. The result of the Kolmogorov–Smirnov test showed that the distribution of the sample does not follow normality in the level of achievement of the cartwheel (Z = 0.201, *p* = 0.001) and the handstand (Z = 0.146, *p* = 0.001).

### 3.1. Cartwheel

For the level of achievement in the execution of the cartwheel (Table 2), a very high percentage of students executed the skill insufficiently throughout the eight academic years (women n = 82, 47.1%; men n = 342, 68.2%). The Mann–Whitney test indicated that the values of achieving the skill was greater for women (n = 93, 54.4%) than men (n = 159, 31.6%) (U = 32.491, *p* = 0.001). A small percentage of students did not take the test because they showed an inability to perform the movement (women n = 11, 6.3%; men n = 36, 7.2%).

Table 3 shows the analysis results of the execution of the cartwheel. We observe that Phases 2 and 4, which are related to alternating hand and foot supports were achieved by a large majority of students. The same is not true of the main phase of movement (Phase 3), which requires a greater technical scope. This phase was achieved by a small group of students, along with Phases 1 and 5, the start and end of the movement related to postural control of the exercise. There was also a group of students who did not execute the skill.

Analyzing the total percentage of the eight academic years and gender (Table 4), after applying the chi^2^ test, it was observed that there were significant differences (*X*^2^ (2, 675) = 55.033, *p* = 0.001) in Phase 3, the main phase of the movement, and women were the best performers.

### 3.2. Handstand

In classifying the level of achievement in the execution of the handstand (Table 5), we can see that over the period analyzed, a very small percentage of students executed the skill with more than five points out of 10. Segregating the data by gender, we note that, over the eight academic years, men had a lower percentage (n = 116, 23.1%) than women (n = 62, 36.1%) for total achievement. A very high percentage of students executed the skill insufficiently throughout the eight academic years (women n = 90, 51.7%; men n = 324, 64.7%), with a significant difference (U = 37.970, *p* = 0.019; *p* ≤ 0.05). There was a small percentage of students who did not perform the test because they were unable to perform the movement (women n = 22, 12.6%; men n = 61, 12.1%).

Analyzing the results obtained for the execution of a handstand by movement phase (Table 6), we observe that Phase 2 (support of hands with separation by the width of the shoulders) was performed correctly by a large percentage of students. Phase 6 (alternate leg reception), which refers to movement recovery, was achieved by about half of the students. The other phases, which relate to more technical aspects of execution such as postural control and strength, were executed correctly by a smaller percentage of students. There was a small number of students who did not perform this skill.

Data on the total percentage of the eight academic years analyzed by phase and gender (Table 7) show that there are significant differences (*X*^2^(3, 675) = 21.812, *p* = 0.001) in Phase 4 (extended body alignment of the arm–trunk–legs segments) of 0.04 (*p* ≤ 0.05) and in Phase 6 (alternate leg reception) are significant differences (*X*^2^(2, 675) = 9.280, *p* = 0.010) of 0.024 (*p* ≤ 0.05), with women getting the best results.

## 4. Discussion

The objective of this study was to analyze gender differences in the gymnastic competence of first-year SPAS students prior to their university education through an initial evaluation process that there were from eight academic years different.

The overall results obtained for both genders over almost a decade show a trend of poor and insufficient level of technical achievement of the selected skills. Although the acrobatic skills evaluated are considered to be within the basics and can be developed naturally and through practice at an early age, the minimal development of gymnastic competition of young people during their schooling years remains evident. In this sense, Davis [22] argued that gymnastics skills are developed inappropriately or, more directly, are not developed at all because of the lack of training of students in gymnastics, due to the lack of understanding of educational gymnastics as an area of knowledge or the lack of training of physical education teachers to teach gymnastics in different contexts (educational, recreational and competitive). This situation can lead to a lack of skill by students training in physical education when the time comes to develop such contents, since previously they did not work on them or did so insufficiently.

In relation to gender, we note that the deficiencies were detected in the analysis of the level of acrobatic achievement; men were less competent than women in both skills, the cartwheel and the handstand. In the case of the cartwheel, the most deficient phase for men was the one that involved body extension, highlighting the alignment and extension of the legs at the moment of balance and turning with the legs open in a lateral spagat, typical of this activity, indicating that the flexibility of these segments is poor for men. As pointed out by Delaš et al. [23], there is a positive correlation between better flexibility and correct lateral cartwheel execution. In this vein, Lopez et al. [24] determined that women have better flexibility than men and this may be related to genetic, structural and/or environmental factors. As for the handstand, the movements that require coordination and stability in the descent phases of the activity, with a separation of the legs in a spagat completely extended with a slow and controlled movement, are also less developed in men. A study [25] indicated that boys have less postural control and balance than girls. These findings reinforce the possible gender differences in fundamental motor skills, with men showing better fundamental motor skills such as quick motions and jumping, and women better flexibility, agility and balance [26,27], qualities that at the ages of 5–6 are similarly developed [28,29].

Another factor to note, and that may be related to the low levels of achievement by students of both genders, is the poor physical condition of adolescents [30,31]. These deficiencies could influence the insufficient performance of the acrobatic movements analyzed.

Gymnastic skills can be complex, and most require a high level of neuromuscular coordination for execution and longer practice time and experience to be acquired [5]. Gymnastic skills need time for training and preparation, and, according to the time available in the school year, it can make it difficult to teach [32]. However, it is shown that gymnastics in general could be a tool to improve the motor skills of children and adolescents, as evidenced by various studies [22,33,34], but it requires a lasting approach over time, an issue that does not seem to be addressed in reality.

Our study has some limitations, since some variables were not taken into account, such as the type of center where the secondary school students carried out their studies, the students’ previous experience with these abilities, and their affinity with or interest in this type of content.

As a future line of study, we propose mainly to investigate the possible causes that could generate these deficiencies in the learning of gymnastic skills. In addition, considering the role of physical education teachers in these issues will be fundamental for the analysis of this problem.

## 5. Conclusions

This study contributes to the literature in the field of gymnastic and acrobatic skills with a study of the acquisition of gymnastic skills in stages over eight years prior to university and the influence of gender. The results show that in the initial evaluation, the level of execution of cartwheel and handstand skills is insufficient in both genders. However, there are significant differences in favor of girls in some stages that require greater flexibility and postural control.

This trend was maintained throughout the eight academic years analyzed, showing us an educational reality, where in pre-university training the content associated with basic gymnastic and acrobatic skills is not well developed in physical education curriculums. This subject should provide the opportunity to integrate motor tasks with different levels of complexity progressively from the early ages to adolescence, as well as the attitudes and emotions associated with motor behavior and individual interests, thus allowing it to be formed integrally and according to their needs.

## Figures and Tables

**Table 1:** Distribution of students throughout the academic years analyzed, according to gender.

Academic Years	2010/11	2011/12	2012/13	2013/14	2014/15	2015/16	2016/17	2017/18	Total
**Participants**	87	79	85	87	87	87	87	76	675
**Women**	21	18	28	26	21	20	21	19	174
**Men**	66	61	57	61	66	67	66	57	501

**Table 2:** Level of achievement of cartwheel, according to academic years, temporary totality and gender.

Level	2010/11		2011/12		2012/13		2013/14		2014/15		2015/16		2016/17		2017/18		Total	
	W	M	W	M	W	M	W	M	W	M	W	M	W	M	W	M	W	M
Ne	9.5	4.5	11.1	1.6	0.0	0.0	0.0	3.3	0.0	1.5	10.0	16.4	14.3	18.2	10.5	10.5	6.3	7.2
Vp	0.0	6.1	5.6	8.2	14.3	0.0	0.0	3.3	9.5	3.0	0.0	1.5	0.0	1.5	5.3	1.8	5.2	3.2
P	14.3	16.7	16.7	11.5	14.3	14.0	0.0	18.0	4.8	12.1	5.0	9.0	4.8	13.6	10.5	15.8	8.6	13.8
Ins	52.4	51.5	27.8	40.9	0.0	64.9	30.8	40.9	23.8	45.5	35.0	31.3	23.8	34.9	21.1	47.4	25.8	44.3
En	0.0	6.1	16.7	14.8	0.0	7.0	0.0	1.6	4.8	7.6	5.0	16.4	19.0	19.7	0.0	8.8	5.2	10.4
Nb	0.0	3.0	5.6	4.9	17.9	0.0	0.0	1.6	0.0	3.0	0.0	0.0	0.0	3.0	0.0	0.0	3.4	2.0
Rm	14.3	4.5	5.6	11.5	0.0	7.0	50.0	19.7	33.3	18.2	30.0	16.4	23.8	4.5	47.4	12.3	25.3	11.8
Os	9.5	7.5	11.1	6.6	53.6	7.7	15.3	11.4	23.8	9.0	15.0	9.0	14.3	4.5	5.3	3.5	20.1	7.4

W, women; M, men; Ne, not execute; Vp, very poor; P, poor; Ins, insufficient; En, enough; Nb, not bad; Rm, remarkable; Os, outstanding.

**Table 3:** Analysis of technical execution of cartwheel, according to movement phase and gender.

Women:	Phase 1		Phase 2		Phase 3		Phase 4		Phase 5		Not Execute
	√	X	√	X	√	X	√	X	√	X	
2010/11	9.5	81.0	90.5	0.0	23.8	66.7	71.4	19.0	14.3	76.2	9.5
2011/12	27.8	61.1	83.3	5.6	16.7	72.2	66.7	22.2	11.1	77.8	11.1
2012/13	25.0	75.0	85.7	14.3	71.4	28.6	53.6	46.4	53.6	46.4	0.0
2013/14	11.5	88.5	96.2	3.8	65.4	34.6	96.2	3.8	7.7	92.3	0.0
2014/15	28.6	71.4	81.0	19.0	61.9	38.1	85.7	14.3	14.3	85.7	0.0
2015/16	10.0	80.0	90.0	0.0	45.0	45.0	85.0	5.0	15.0	75.0	10.0
2016/17	14.3	71.4	85.7	0.0	38.1	47.6	76.2	9.5	28.6	57.1	14.3
2017/18	5.3	84.2	84.2	5.3	52.6	36.8	73.7	15.8	0.0	89.5	10.5
**Men:**											
2010/11	16.7	78.8	86.4	9.1	12.1	83.3	75.8	19.7	7.6	87.9	4.5
2011/12	27.9	70.5	90.2	8.2	18.0	80.3	77.0	21.3	8.2	90.2	1.6
2012/13	8.8	91.2	100	0,0	14.0	86.0	63.2	36.8	28.1	71.9	0.0
2013/14	9.8	86.9	91.8	4.9	32.8	63.9	73.8	23.0	6.6	90.2	3.3
2014/15	16.7	81.8	95.5	3.0	28.8	69.7	80.3	18.2	9.1	89.4	1.5
2015/16	7.5	76.1	82.1	1.5	25.4	58.2	73.1	10.4	19.4	64.2	16.4
2016/17	16.7	65.2	75.8	6.1	12.1	69.7	59.1	22.7	21.2	60.6	18.2
2017/18	14.0	75.4	86.0	3.5	15.8	73.7	71.9	17.5	0.0	89.5	10.5

√, correct; X, wrong.

**Table 4:** Gender differences in achievement level of cartwheel over eight academic years.

Gender	Phase 1		Phase 2		Phase 3 *		Phase 4		Phase 5		Not Execute
	√	X	√	X	√	X	√	X	√	X	
Women	**16.7**	**77.0**	**87.4**	**6.3**	**48.9**	**44.8**	**75.9**	**17.8**	**19.5**	**74.1**	6.3
Men	14.8	78.0	88.2	4.6	20.0	72.9	71.9	21.0	12.6	80.2	7.2

√, correct; X, wrong. * Significant difference (*p* ≤ 0.05).

**Table 5:** Level of achievement of handstand according to academic years, temporary totality and gender.

Level	2010/11		2011/12		2012/13		2013/14		2014/15		2015/16		2016/17		2017/18		Total	
	W	M	W	M	W	M	W	M	W	M	W	M	W	M	W	M	W	M
Ne	23.8	7.6	16.7	4.9	0.0	0.0	3.8	18.0	19.0	9.1	15.0	23.9	14.3	10.6	15.8	22.8	12.6	12.2
Vp	14.3	10.6	5.6	9.8	14.3	0.0	0.0	1.6	9.5	0.0	0.0	3.0	0.0	1.5	0.0	0.0	5.7	3.4
P	33.3	25.8	33.4	46.0	46.4	85.9	23.1	32.4	9.5	30.3	20.0	25.4	23.8	36.4	10.5	21.1	25.9	37.4
Ins	14.3	36.4	16.7	18.1	0.0	7.0	38.5	19.7	14.3	21.2	10.0	23.9	33.3	36.4	31.6	28.1	19.5	24.2
En	9.5	10.6	0.0	4.9	0.0	0.0	3.8	1.6	0.0	1.5	10.0	7.5	9.5	4.5	36.8	24.6	8.0	6.8
Nb	4.8	7.6	16.7	6.6	0.0	7.0	7.7	4.9	4.8	4.5	20.0	6.0	4.8	3.0	0.0	0.0	6.9	5.0
Rm	0.0	0.0	11.2	8.2	0.0	0.0	11.5	9.9	14.3	19.7	10.0	7.5	0.0	4.5	5.3	3.5	6.3	6.8
Os	0.0	1.5	0.0	1.6	39.3	0.0	11.5	11.5	28.5	13.6	15.0	3.0	14.3	3.0	0.0	0.0	14.9	4.4

W, women; M, men; Ne, not execute; Vp, very poor; P, poor; Ins, insufficient; En, enough; Nb, not bad; Rm, remarkable; Os, outstanding.

**Table 6:** Analysis of technical execution of handstand according to movement phases and gender.

Women:	Phase 1		Phase 2		Phase 3		Phase 4		Phase 5		Phase 6		Not Execute
	√	X	√	X	√	X	√	X	√	X	√	X	
2010/11	4.8	71.4	52.4	23.8	28.6	47.6	9.5	66.7	0.0	76.2	28.6	47.6	23.8
2011/12	22.2	61.1	77.8	5.6	22.2	61.1	33.3	50.0	5.6	77.8	66.7	16.7	16.7
2012/13	39.3	60.7	85.7	14.3	39.3	60.7	39.3	60.7	39.3	60.7	53.6	46.4	0.0
2013/14	19.2	76.9	92.3	3.8	65.4	30.8	30.8	65.4	15.4	80.8	80.8	15.4	3.8
2014/15	47.6	33.3	71.4	9.5	61.9	19.0	42.9	38.1	33.3	47.6	42.9	38.1	19.0
2015/16	35.0	50.0	85.0	0.0	60.0	25.0	45.0	40.0	25.0	60.0	60.0	25.0	15.0
2016/17	9.5	76.2	85.7	0.0	61.9	23.8	23.8	61.9	14.3	71.4	61.9	23.8	14.3
2017/18	68.4	15.8	84.2	0.0	47.4	36.8	5.3	78.9	0.0	84.2	84.2	0.0	15.8
**Men:**													
2010/11	39.4	53.0	37.9	54.5	53.0	39.4	15.2	77.3	4.5	87.9	42.4	50.0	7.6
2011/12	37.7	57.4	75.4	19.7	21.3	73.8	16.4	78.7	9.8	85.2	47.5	47.5	4.9
2012/13	0.0	100	100	0.0	14.0	86.0	7.0	93.0	0.0	100	56.1	43.9	0.0
2013/14	21.3	60.7	77.0	4.9	39.3	42.6	21.3	60.7	23.0	59.0	47.5	34.4	18.0
2014/15	36.4	54.5	90.9	0.0	56.1	34.8	27.3	63.6	28.8	62.1	51.5	39.4	9.1
2015/16	13.4	62.7	65.7	10.4	44.8	31.3	10.4	65.7	13.4	62.7	38.8	37.3	23.9
2016/17	4.5	84.8	87.9	1.5	48.5	40.9	15.2	74.2	9.1	80.3	37.9	51.5	10.6
2017/18	50.9	26.3	75.4	1.8	36.8	40.4	1.8	75.4	3.5	73.7	57.9	19.3	22.8

√, correct; X, wrong.

**Table 7:** Gender differences in achievement level of handstand over eight academic years.

Gender	Phase 1		Phase 2		Phase 3		Phase 4 *		Phase 5		Phase 6 *		Not Execute
	√	X	√	X	√	X	√	X	√	X	√	X	
Women	30.5	56.9	79.9	7.5	48.9	38.5	29.3	58.0	17.8	69.5	59.8	27.6	12.6
Men	25.5	62.5	75.8	12.0	39.9	47.9	13.8	73.3	11.8	76.0	47.1	40.7	12.2

√, correct; X, wrong. * Significant difference (*p* ≤ 0.05).
